# Religiosity, Impulsivity, and Compulsivity in University Students

**DOI:** 10.1017/S1092852922000815

**Published:** 2022-05-20

**Authors:** Jon E. Grant, Katherine Lust, Samuel R. Chamberlain

**Affiliations:** 1 Department of Psychiatry & Behavioral Neuroscience University of Chicago, Chicago, IL, USA; 2 Boynton Health Service, University of Minnesota USA; 3 Department of Psychiatry, Faculty of Medicine, University of Southampton, UK; and Southern Health NHS Foundation Trust, Southampton, UK

**Keywords:** religiosity, spirituality, addiction, impulsivity

## Abstract

**Background:**

Prior research suggests that religiosity may be associated with higher levels of mental health in certain domains (e.g. self-esteem, rates of substance use problems). However, very little is known about religiosity and impulsive plus compulsive tendencies. This study examined associations between religiosity and impulsive and compulsive behaviors and traits among university students.

**Methods:**

9,449 students received a 156-item anonymous online survey which assessed religiosity, alcohol and drug use, mental health issues, and impulsive and compulsive traits. Two groups of interest were defined: those with high religiosity, and those with low religiosity, based on z-scores. The two groups were compared on the measures of interest.

**Results:**

3,572 university students (57.1% female) responded to the survey. Those with high levels of organizational religious activity (ORA), as well as those with high levels of intrinsic or subjective religiosity (IR) differed from their fellow students in having better self-esteem, being less likely to have alcohol or drug problems, and generally being less impulsive in terms of attention and planning. These associations were of small effect size except for the link between religiosity and lower impulsivity, which was of medium effect size.

**Conclusion:**

This study shows a link between higher religiosity and lower impulsivity, as well as higher levels of mental health across several domains. Whether these associations are causal – and if so, the direction of such causality – requires rigorous longitudinal research.

## Introduction

Religion has had an enduring impact on human society and has shaped how countless people perceive themselves and their world (1). Although the evolutionary basis of religion continues to be debated, some conceptual approaches view religion as either a byproduct of fundamental cognitive processes or as an adaptive social system designed to promote cooperation and other prosocial behaviors (2). Given that religion appears to have adaptive value, it is perhaps unsurprising that most studies support a positive association between religiosity and mental health (3,4). The behavioral mechanisms that may explain these findings across diverse cultures, however, are a matter of controversy (5).

Religiosity has also shown some association with spirituality, but there are differences between these constructs. Whereas religion represents a socially-organized system of beliefs (13), spirituality is usually defined by the person and often refers to a person’s sense of meaning in life and a connection to a power greater than the self. Studies in the field of addictions have suggested that both religiosity and spirituality often increase self-control over unhealthy behaviors by giving people a feeling of purpose, reinforcing core values, and promoting cognitive changes (14–16).

According to an emerging body of evidence, the religion/health relationship may be partly explained by the concept of self-control (6–8). Self-control is a construct linked to several distinct cognitive processes and personality traits, including conscientiousness (an index of one’s tendency to be organized, responsible, and hard-working) and the ability to delay immediate gratification. By these measures, the more religious a person is the greater the capacity for self-control, on average, compared to non-religious counterparts (7–10). Indeed, self-control is thought to be a crucial element of religious practice—consider that virtually all religions require their members to participate in effortful ritual practices or behaviors, such as public prayer or fasting, that require the exercise of self-control (7,8). In turn, self-control may promote greater subjective psychological well-being (11) or mediate the relationship between religiosity and health behaviors (e.g., substance use) (12). Notably, studies of diverse social groups have found that subjective well-being is better predicted by involvement in institutional religious practices (e.g., attendance at religious services) than by private religious practice or personal religious belief (8). Private or subjective forms of religiosity, however, may preferentially benefit some clinical populations (8).

Relevant to the social construct of ‘self-control’ are the concepts, from the neurosciences, of impulsivity and compulsivity. Impulsivity refers to the tendency towards hasty or poorly thought out actions, leading to untoward actions (cite); whereas compulsivity is the tendency towards repetitive habitual actions that persist despite consequent functional impairment (cite). These two processes contribute at different stages in the progression from a potentially risky act (e.g. drinking alcohol or gambling) through to getting ‘stuck’ in these behaviors. Impulsivity and compulsivity can be fruitfully measured using convenient self-report questionnaires (cite).

Young adulthood is a time where many individuals engage in and struggle with controlling unhealthy behaviors, but little is known about the influences of religiosity in this age cohort. Our study examined links between impulsivity, compulsivity, and multiple dimensions of religiosity in a large sample of university students using a voluntary, anonymous internet-based survey. We hypothesized that religiosity (specifically, the frequency of religious behaviors performed in a group or social setting) would be associated with lower trait impulsivity, lower trait compulsivity, and lower rates of non-substance or “behavioral” addictions (gambling disorder, compulsive sexual behavior, binge eating disorder) (17).

## Methods

### Survey Design

The Department of Psychiatry and Behavioral Neuroscience at the University of Chicago and Boynton Health at the University of Minnesota jointly developed the *Health and Addictive Behaviors Survey* to assess mental health and well-being in a large sample of university students. The survey included basic demographics as well as questions from a number of validated screening tools examining mental health and psychological well-being. All study procedures were carried out in accordance with the Declaration of Helsinki and were approved by the Institutional Review Board of the University of Minnesota.

### Participants

A sub-sample of 10,000 college and graduate students at a large, nondenominational and coeducational Midwestern university were chosen by random, computer-generated selection from a total pool of approximately 60,000 students at the university. The survey was distributed over a three-week period during the fall 2016 semester, with invitations sent by email and surveys completed online. Of the 10,000 email invitations, 9449 were successfully received by the recipients (i.e., did not bounce back). Recipients of the email were first required to view the IRB-approved online informed consent page, at which point students could choose to participate in the survey or opt out. The survey asserted that all information was both anonymous and confidential. Compensation was offered at the conclusion of the survey by randomly selecting respondents to receive tablet computers (*n* = 3) or gift certificates to an online retailer in the amounts of $250 (*n* = 4), $500 (*n* = 2), and $1000 (*n* = 1). Participants were required to review all survey questions to be eligible for prize drawings, but were not required to answer any question they did not wish to answer. Of the 9449 students who received the invitation to participate, 3659 (38.7%) completed the survey.

### Assessments

The self-report survey consisted of 156 questions and took participants approximately 30 minutes to complete. Survey questions assessed demographic information (including religious affiliation), sexual behavior, self-reported academic achievement (i.e., grade point average [GPA]), and clinical characteristics of participants, including levels of mental health and substance use.

In order to assess other aspects of mental health function and religiosity, participants were also asked to complete the following measures:

Religiosity was assessed using the *Duke University Religion Index (DUREL)*. The DUREL is a valid and reliable, 5-item measure of religious involvement across three domains: organizational religious activity (ORA), non-organizational religious activity (NORA), and intrinsic or subjective religiosity (IR) (18). The ORA domain assesses frequency of participation in religious services (1 = *never* to 6 = *more than once/week*). The NORA domain measures the extent of involvement in private religious activities, such as prayer or the study of religious texts (1 = *rarely or never* to 6 = *more than once a day*). The IR domain (3 items) assesses the degree to which the participant is motivated by or committed to his or her religion (1 = *definitely not true* to 5 = *definitely true of me*). Higher scores reflect greater religiosity. The DUREL demonstrated good internal consistency in our sample (Cronbach α = 0.924).

Putative disorders of impulse control were screened for using the *Minnesota Impulsive Disorders Interview (MIDI)*. In this study, we focused on gambling disorder and compulsive sexual behavior (19–20).

Alcohol use behaviors and related problems were assessed using the *Alcohol Use Disorders Identification Test (AUDIT)*. Each item is scored 0-4, with a maximum of 40 points possible. A score of 8 or greater indicates hazardous or harmful alcohol use (21).

Problematic substance use was identified using the *Drug Abuse Screening Test (DAST-10)*. A score of 3 is used to screen for a drug use disorder (22–23).

Depressive symptoms were measured using the *Patient Health Questionnaire (PHQ-9)*. The PHQ-9 is based directly on DSM-IV-TR criteria for major depressive disorder (24).

Posttraumatic stress disorder (PTSD) was screened for using the *Primary Care PTSD Screen (PC-PTSD)*. The PC-PTSD is based on DSM-IV PTSD criteria (25). A score of ≥3 indicates probable PTSD.

Generalized anxiety disorder (GAD) was screened for using the *Generalized Anxiety Disorder 7 (GAD-7)*. Total scores of 10 or greater indicate clinically significant anxiety (26).

Attention-deficit/hyperactivity disorder (ADHD) was screened for using the *Adult ADHD Self-Report Scale (ASRS-v1.1)*. The ASRS has demonstrated strong psychometric properties (27).

Global feelings of self-worth or self-regard were measured using the *Rosenberg Self-Esteem Scale (RSES)*. Scores below 15 suggest low self-esteem (28).

Impulsivity was assessed using the *Barratt Impulsiveness Scale, Version 11 (BIS-11)*. The BIS-11 is a 30-item measure designed to assess impulsivity across three dimensions: attentional (inability to concentrate), motor (acting without thinking), and non-planning (lack of future orientation) (29–30). Each of the 30 items is rated on a 4-point scale of 1 (*rarely/never*) to 4 (*almost always*), where 4 indicates greater impulsiveness.

Compulsive traits were measured using the Cambridge–Chicago Compulsivity Trait Scale (CHI-T). The scale has shown excellent psychometric properties, with high internal consistency (Cronbach’s alpha = 0.8), excellent convergent validity against gold-standard assessments for a variety of compulsive disorders (each *p* < .001 for gambling disorder, obsessive-compulsive disorder, and substance use disorder symptoms), and excellent discriminant validity against other constructs such as depression (31). The validity of the CHI-T has also been confirmed in a large sample of the general population and in people with mental health and psychiatric diagnoses (32).

### Data Analysis

Only respondents with complete data on at least one the DUREL subscales were included in the analyses (*N* = 3564; 99.8%). Total scores for each of the three subscales were transformed to standardized *z* scores and participants were categorized based on level of religiosity: low (*z*<−1) or high (*z*>+1). Participants not scoring in these ranges played no further role in the analysis. Distributions of the third score (NOR) did not permit this approach and so we focused on the two where we had adequately large samples of folks with low and high. Groups were compared on demographic and clinical measures using independent sample *t* tests for continuous variables (or equivalent nonparametric tests, as indicated in the text) and chi-square tests for categorical variables. Effect sizes were calculated for all significant differences, which were determined for Likelihood ratio test using Cramer’s V (V = 0.1 is considered a small effect size, 0.3 is medium, and 0.5 is large) (28). Continuous variables were tested for statistical difference using F-test and Cohen’s d for effect size, effect sizes of 0.02, 0.15, and 0.35 are termed *small*, *medium*, and *large*, respectively (32). SPSS was used for all statistical analyses (version 25; IBM). Statistical significance was defined as *p* ≤ 0.01 to account for multiple comparisons.

## Results

The demographic characteristics of the 3,572 participants (57.1% female) are presented in Table 1. Overall, the mean ORA score was 2.52 (1.49), mean NORA score was 1.98 (1.51) and the mean IRA score was 7.66 (4.17). Participants with high ORA and high IR showed the same patterns in terms of demographics. That is, they were more likely to be female, married or engaged, and identify as Catholic, Muslim, protestant, or “other Christian” than those low on ORA or IR. Grade point average did not differ based on religiosity.

In terms of mental health, the data are presented in Table 2. Participants who scored high on ORA and IR were significantly less likely to have alcohol or drug problems and less likely to have low self-esteem. In addition, those with high ORA were significantly less likely to screen positive for PTSD.

Finally, although participants with higher levels of ORA and IR did not differ significantly on a measure of compulsivity from those with lower levels, they did significantly differ in two domains of impulsiveness, attentional and non-planning impulsiveness (Table 3).

## Discussion

This study examined two aspects religiosity and their links with mental health with a particular focus on impulsive and compulsive tendencies. The two aspects of religiosity examined were organizational religiosity (propensity to attend and engage with formal religious services) and intrinsic religiosity (propensity to integrate religion into one’s life endeavors) (cite). We focused on a large sample of university students and the possible associations between religiosity and a range of demographic/clinical measures, and questionnaire-based measures of impulsivity. We found that students who scored high on either types of religiosity were less impulsive, had better self-esteem and were less likely to have alcohol or drug problems. These results seem generally in keeping with previous examinations of religiosity in young adults. In a previous study using the DRUEL in a small sample of 93 patients with mental illness who had attempted suicide and 61 healthy individuals, Caribe and colleagues (2015) found that the healthy individuals scored higher scores in the religiosity domains and this was associated with lower scores on the BIS impulsiveness scale. Similarly, a study of 448 students in Iran found that those who engaged more often in organized religious activities and had higher intrinsic religiosity were less likely to engage in risky behaviors such as sexual risk taking, careless driving, violence, smoking, as well as alcohol and drug abuse (Ameri et al., 2017).

The links between religiosity and other measures in the current study were generally of small effect size, which would be in keeping with prior cross-sectional research in other areas of mental health (cites) including more recent longitudinal work (cite). However, the one finding in this study that demonstrated a moderate effect size was that higher religiosity was associated with less attentional impulsiveness. This BIS subscale reflects a tendency to have rapid shifts in attention, to have difficulties in task focus, and to become impatient with complexity.

The fact that religiosity was not associated with compulsivity is a novel finding, contrary to our predictions, and is in contrast to the link found with impulsivity. These results may suggest that people with high religiosity are less likely to engage in impulsive acts on the spur of the moment (e.g. early stages of alcohol use or gambling), but are just as likely to develop habitual repetitive behaviors over time after initially engaging in these activities. It is interesting to consider how this may reflect the focus of several mainstream religions on often complete avoidance of certain addictive substances and behaviors (e.g. alcohol, gambling). Does this reflect our innate tendency to develop habits irresepective of religiosity, whereas avoiding early stages of potentially problematic behavior is something we are abler to do and this is aided by religious frameworks?

In terms of mental health problems, we found that higher levels of religiosity were significantly associated with higher self-esteem and, in the case of organized religion, with lower levels of PTSD. Thus our findings add to growing evidence of the potential small effect size protective factors of religiosity in young people. A study of Veterans similarly found that PTSD was less likely in those with greater religiosity (Sharma et al., 2017). This finding could be explained by the sense of purpose and community that organized religion instills in some people, or it could be an indirect effect. That is, those with higher organizational religiosity also had better self-esteem, were less impulsive and less likely to have alcohol and drug problems. Given that PTSD has been associated with alcohol and drug problems (Panza et al., 2022), and that less impulsive people may be less likely to have traumatic situations (Santos et al., 2022), multiple interacting variables may explain the lower rates of PTSD in those who are more religious.

This study of religiosity in young adults has the advantage of being relatively large. Nonetheless, there are several limitations that should be considered. The study was cross-sectional and hence the direction of causality of any effects cannot be established – this would require longitudinal research on the topic; however, we hope that such cross-sectional data will encourage such follow-up. Given that associations were generally of small effect size, we did not attempt to examine mediation between variables. There are limitations inherent in the study being conducted using an online interface via the Internet – diagnostic assessment may be less accurate via such an online survey compared to in-person assessment by a clinician; there may be responder biases; and there may be under-reporting (though this possibility is reduced by individuals’ responses not being lacked to personally identifiable information). Our splitting of the sample into those with high and low religiousity was a useful way of presenting the data since it is intuitive to the reader; however of course there are other ways of operationalizing high and low religiosity that could be used.

In summary, we found that higher levels of religiosity in university students were associated with lower rates of impulsivity (medium effect size) as well as relatively higher levels of mental health (small effect size), but not with different levels of compulsivity. Whether religiosity leads to being less impulsive or vice versa, both, or the link can be accounted for by other variables, remains unclear. The link with impulsive traits may indicate less propensity of people with high religiousity to spontaneously undertake or engage with potentially harmful activities (e.g. alcohol or gambling) but that once initiated, there is a similar tendency to get stuck in a given habitual pattern as compared to people with low levels of religiosity.

## Figures and Tables

**Table 1:** Demographics of university students based on level of religiosity^a^

	Organizational religious activity		Statistic	Intrinsic religiosity		Statistic
	Z Score <-1.00 N=1338	Z Score >1.00 N=450		Z Score <-1.00 N=958	Z Score >1.00 N=867	
Gender						
Male	516(41.1)	170(40.3)	LR=11.789 df=2 P=.003 V=.073	373(41.8)	261(32.2)	LR=29.093 df=2 P<.001 V=.129
Female	704(56.0)	250(59.2)		494(55.4)	543(67.0)	
Transgender, genderqueer, or alternative descriptor	37(2.9)	2(0.5)		25(2.8)	7(0.9)	
						
Religious affiliation						
Agnostic	352(26.3)	1(0.2)	LR=1129.31 df=11 P=.000 V=.770	240(25.1)	8(0.9)	LR=1573.25 df=11 P<.001 V=.829
Atheist	369(27.6)	2(0.4)		358(37.4)	4(0.5)	
Buddhist	17(1.3)	2(0.4)		11(1.1)	11(1.3)	
Catholic	44(3.3)	100(22.2)		27(2.8)	172(19.8)	
Hindu	13(1.0)	2(0.4)		1(0.1)	16(1.8)	
Jewish	8(0.6)	3(0.7)		12(1.3)	9(1.0)	
Muslim	12(0.9)	28(6.2)		1(0.1)	51(5.9)	
Protestant	8(0.6)	123(27.3)		2(0.2)	193(22.3)	
Other Christian	61(4.6)	144(32.0)		13(1.4)	289(33.3)	
Other	119(8.9)	2(0.4)		56(5.8)	19(2.2)	
Chose more than one religion	225(16.8)	39(8.7)		172(18.0)	83(9.6)	
Prefer to not answer	110(8.2)	4(0.9)		65(6.8)	12(1.4)	
						
Student Status						
Undergraduate	885(66.1)	290(64.4)	LR=1.00 df=2 P=.606 V=.024	640(66.8)	548(63.2)	LR=4.976 df=2 P=.083 V=.052
Graduate/Professional	446(33.3)	156(34.7)		315(32.9)	311(35.9)	
Non-degree seeking	7(0.5)	4(0.9)		3(0.3)	8(0.9)	
						
Race/ethnicity, Caucasian	913(72.7)	282(66.7)	LR=5.604 df=1 P=.018 V=.058	685(76.8)	599(73.8)	LR=2.093 df=1 P=.148 V=.035
						
Relationship Status						
Single	578(43.2)	231(51.3)	LR=62.905 df=3 P=.000 V=.186	394(41.1)	414(47.8)	LR=59.713 df=3 P<0.001 V=.180
Dating	578(43.2)	110(24.4)		441(46.0)	265(30.6)	
Engaged/married	169(12.6)	106(23.6)		111(11.6)	182(21.0)	
Other	13(1.0)	3(0.7)		12(1.3)	6(0.7)	
						
College GPA						
Below 2.50	26(2.0)	5(1.1)	LR=4.647 df=3 P=.200 V=.051	18(1.9)	11(1.3)	LR=1.435 df=3 P=.697 V=..028
2.50-2.99	120(9.1)	36(8.2)		78(8.2)	69(8.0)	
3.00-3.49	452(34.2)	135(30.6)		325(34.2)	286(33.3)	
3.50-4.00	725(54.8)	265(60.1)		528(55.6)	492(57.3)	

a Measured by the Duke University Religion Index (DUREL) Data refer to *N* (percentage), LR=Likelihhod Ratio, V=Cramer’s V GPA = grade point average

**Table 2:** Mental health problems of university students based on level of religiosity^a^

	Organizational religious activity		Statistic	Intrinsic religiosity		Statistic
	Z Score <-1.00 N=1338	Z Score >1.00 N=450		Z Score <-1.00 N=958	Z Score >1.00 N=867	
PHQ9-Major depression disorder^b^	69(5.4)	12(2.9)	LR=5.078 df=1 P=.024 V=.052	54(6.0)	33(4.1)	LR=3.179 df=1 P=.075 V=.043
PC-PTSD^c^	212(16.6)	44(10.3)	LR=10.821 df=1 P=.001 V=.077	137(15.0)	106(12.9)	LR=1.651 df=1 P=.199 V=.031
Generalized anxiety disorder^d^	232(18.5)	57(13.6)	LR=5.407 df=1 P=.020 V=.056	168(18.6)	130(16.0)	LR=2.005 df=1 P=.157 V=.034
Compulsive sexual behavior	46(3.7)	14(3.3)	LR=0.118 df=1 P=.731 V=.008	32(3.6)	26(3.2)	LR=0.164 df=1 P=.686 V=.010
Binge eating disorder	37(2.9)	8(1.9)	LR=1.440 df=1 P=.230 V=.028	28(3.1)	11(1.3)	LR=6.203 df=1 P=.013 V=.059
ADHD	241(19.1)	62(14.6)	LR=4.602 df=1 P=.032 V=.051	174(19.4)	120(14.8)	LR=6.371 df=1 P=.012 V=.061
Gambling disorder	5(0.4)	0(0.0)	LR=2.895 df=1 P=.089 V=.031	2(0.2)	3(0.4)	LR=0.317 df=1 P=.574 V=.014
Low self-esteem^e^	213(17.1)	45(10.8)	LR=9.965 df=1 P=.002 V=.075	156(17.7)	83(10.5)	LR=18.110 df=1 P<0.001 V=.103
AUDIT score >=8	335(25.3)	36(8.1)	LR=69.653 df=1 P=.000 V=.184	238(25.1)	150(17.5)	LR=15.649 df=1 P<0.001 V=.093
DAST-10 score >=3	142(10.8)	12(2.7)	LR=33.540 df=1 P=.000 V=.124	95(10.1)	43(5.0)	LR=16.558 df=1 P<0.001 V=.095

a Measured by the Duke University Religion Index (DUREL) Data refer to *N* (percentage), LR=Likelihhod Ratio, V=Cramer’s V

ADHD = attention-deficit/hyperactivity disorder; GAD-7 = General Anxiety Disorder-7; PC-PTSD = Primary Care PTSD Screen; PHQ-9 = Patient Health Questionnaire

a Measured by the Duke University Religion Index (DUREL)

b PHQ-9 score ≥10

c PC-PTSD score ≥3

d GAD-7 score ≥10

e RSES score <15

**Table 3:** Impulsivity and compulsivity of university students based on level of religiosity^a^

	Organizational religious activity		Statistic	Intrinsic religiosity		Statistic
	Z Score <-1.00 N=1338	Z Score >1.00 N=450		Z Score <-1.00 N=958	Z Score >1.00 N=867	
Cambridge-Chicago Compulsivity Trait Scale	9.83(13.69)	8.63(13.22)	F(1,1713)=2.506 P=.114 d=.09	9.79(13.56)	8.31(13.20)	F(1,1742)=5.296 P=.021 d=0.11
Barratt Impulsiveness Scale (BIS-11)						
Attentional impulsiveness	16.70(4.08)	15.25(3.83)	F(1,1639)=39.98 P=.000 d=0.36	16.76(4.25)	15.48(3.85)	F(1,1666)=41.42 P=.000 d=0.32
						
Motor impulsiveness	20.46(4.01)	20.02(4.12)	F(1,1642)=3.716 P=.054 d=0.11	20.22(4.01)	20.25(4.14)	F(1,1668)=0.024 P=.876 d=0.008
						
Non-planning impulsiveness	23.29(4.88)	22.21(4.46)	F(1,1634)=15.736 P=.000 d=0.23	23.01(4.88)	22.28(4.74)	F(1,1665)=9.388 P=.002 d=0.15
						

a Measured by the Duke University Religion Index (DUREL) Data refer to Mean (standard deviation), d=Cohen’s d Cambridge–Chicago Compulsivity Trait Scale Barratt Impulsiveness Scale (BIS-11) Attentional impulsiveness Motor impulsiveness Non-planning impulsiveness

## References

[1] Bloom P (2012). Religion, Morality, Evolution. Annu Rev Psychol.

[2] Shaver JH, Purzycki G, Sosis R (2016). The Oxford Handbook of the Study of Religion.

[3] Moreira-Almeida A, Lotufo Neto F, Koenig HG (2006). Religiousness and mental health: a review. Rev Bras Psiquiatr.

[4] Bonelli RM, Koenig HG (2013). Mental disorders, religion and spirituality 1990 to 2010: a systematic evidence-based review. J Relig Health.

[5] George LK, Ellison CG, Larson DB (2002). Target article: Explaining the relationships between religious involvement and health. Psychol Inq.

[6] Koole SL, McCullough ME, Kuhl J, Roelofsma PHMP (2010). Why religion’s burdens are light: from religiosity to implicit self-regulation. Personal Soc Psychol Rev Off J Soc Personal Soc Psychol Inc.

[7] McCullough ME, Willoughby BLB (2009). Religion, self-regulation, and self-control: Associations, explanations, and implications. Psychol Bull.

[8] Wood C (2017). Ritual well-being: toward a social signaling model of religion and mental health. Relig Brain Behav.

[9] Carter EC, McCullough ME, Kim-Spoon J, Corrales C, Blake A (2012). Religious people discount the future less. Evol Hum Behav.

[10] Paglieri F, Borghi AM, Colzato LS, Hommel B, Scorolli C (2013). Heaven can wait. How religion modulates temporal discounting. Psychol Res.

[11] Hofmann W, Luhmann M, Fisher RR, Vohs KD, Baumeister RF (2014). Yes, but are they happy? Effects of trait self-control on affective well-being and life satisfaction. J Pers.

[12] Walker C, Ainette MG, Wills TA, Mendoza D (2007). Religiosity and substance use: test of an indirect-effect model in early and middle adolescence. Psychol Addict Behav J Soc Psychol Addict Behav.

[13] Longshore D, Anglin MD, Conner BT (2008). Are religiosity and spirituality useful constructs in drug treatment research?. J Behav Health Serv Res.

[14] Grodzicki J, Galanter M (2005). Spirituality and addiction. Subst Abus.

[15] Galanter M (2007). Spirituality and recovery in 12-step programs: An empirical model. J Subst Abuse Treat.

[16] Jennings TL, Lyng T, Gleason N, Finotelli I, Coleman E (2021). Compulsive sexual behavior, religiosity, and spirituality: A systematic review. J Behav Addict.

[17] Grant JE, Potenza MN, Weinstein A, Gorelick DA (2010). Introduction to Behavioral Addictions. Am J Drug Alcohol Abuse.

[18] Koenig HG, Büssing A (2010). The Duke University Religion Index (DUREL): A Five-Item Measure for Use in Epidemological Studies. Religions.

[19] Grant JE (2008). Impulse Control Disorders: A Clinician’s Guide to Understanding and Treating Behavioral Addictions.

[20] Odlaug BL, Grant JE (2010). Impulse-Control Disorders in a College Sample: Results From the Self-Administered Minnesota Impulse Disorders Interview (MIDI). Prim Care Companion CNS Disord.

[21] Saunders JB, Aasland OG, Babor TF, de la Fuente JR, Grant M (1993). Development of the Alcohol Use Disorders Identification Test (AUDIT): WHO Collaborative Project on Early Detection of Persons with Harmful Alcohol Consumption--II. Addict Abingdon Engl.

[22] Skinner HA (1982). The drug abuse screening test. Addict Behav.

[23] Yudko E, Lozhkina O, Fouts A (2007). A comprehensive review of the psychometric properties of the Drug Abuse Screening Test. J Subst Abuse Treat.

[24] Kroenke K, Spitzer RL, Williams JB (2001). The PHQ-9: validity of a brief depression severity measure. J Gen Intern Med.

[25] Prins A, Ouimette P, Kimerling R, Cameron RP, Hugelshofer DS, Shaw-Hegwer J (2003). The primary care PTSD screen (PC-PTSD): Development and operating characteristics. Prim Care Psychiatry.

[26] Spitzer RL, Kroenke K, Williams JBW, Löwe B (2006). A brief measure for assessing generalized anxiety disorder: the GAD-7. Arch Intern Med.

[27] Kessler RC, Adler L, Ames M, Demler O, Faraone S, Hiripi E (2005). The World Health Organization Adult ADHD Self-Report Scale (ASRS): a short screening scale for use in the general population. Psychol Med.

[28] Rosenberg M (1965). Society and the adolescent self-image.

[29] Patton JH, Stanford MS, Barratt ES (1995). Factor structure of the Barratt impulsiveness scale. J Clin Psychol.

[30] Stanford MS, Mathias CW, Dougherty DM, Lake SL, Anderson NE, Patton JH (2009). Fifty years of the Barratt Impulsiveness Scale: An update and review. Personal Individ Differ.

[31] Chamberlain SR, Grant JE (2017). Initial validation of a transdiagnostic compulsivity questionnaire: The Cambridge-Chicago Compulsivity Trait Scale.

[28] Cohen J (1988). Statistical Power Analysis for the Behavioral Sciences.

[31] Tiego J, Trender W, Hellyer PJ, Grant JE, Hampshire A, Chamberlain SR (2022). Measuring compulsivity as a self-reported multi-dimensional transdiagnostic construct: Large-scale (N=182,000) validation of the Cambridge-Chicago Compulsivity Trait Scale. Submitted.

[R34] Caribé AC, Rocha MF, Junior DF, Studart P, Quarantini LC, Guerreiro N, Miranda-Scippa Â (2015). Religiosity and Impulsivity in Mental Health: Is There a Relationship?. J Nerv Ment Dis.

[R35] Ameri Z, Mirzakhani F, Nabipour AR, Khanjani N, Sullman MJM (2017). The Relationship Between Religion and Risky Behaviors Among Iranian University Students. J Relig Health.

[R36] Panza KE, Kline AC, Na PJ, Potenza MN, Norman SB, Pietrzak RH (2022). Epidemiology of DSM-5 alcohol use disorder in U.S. military veterans: Results from the National Health and Resilience in Veterans Study. Drug Alcohol Depend.

[R37] Santos LL, Netto LR, Cavalcanti-Ribeiro P, Pereira JL, Souza-Marques B, Argolo F, Lira SB, Fontes G, Moreira EC, Anthony JC, Koenen KC (2022). Trauma, Anxiety Disorders Study Group-TADSG. Drugs age-of-onset as a signal of later post-traumatic stress disorder: Bayesian analysis of a census protocol. Addict Behav.

[R38] Sharma V, Marin DB, Koenig HK, Feder A, Iacoviello BM, Southwick SM, Pietrzak RH (2017). Religion, spirituality, and mental health of U.S. military veterans: Results from the National Health and Resilience in Veterans Study. J Affect Disord.

